# Impact of R0/R1 Margin Status on Overall Survival in Node-Positive Resected Pancreatic Ductal Adenocarcinoma: Meta-Analysis

**DOI:** 10.1245/s10434-026-19850-4

**Published:** 2026-07-08

**Authors:** A. M. Bonomi, S. Granieri, R. M. Montorsi, E. Gjoni, O. R. Busch, A. A. Vidal-Itriago, J. I. Erdmann, M. G. Besselink

**Affiliations:** 1 Department of Surgery, ASST Brianza-Vimercate Hospital, Vimercate, Italy; 2 Amsterdam UMC, Department of Surgery, location University of Amsterdam, Amsterdam, the Netherlands; 3 Cancer Center Amsterdam, Amsterdam, the Netherlands; 4 Acute care and Trauma Surgery, ASST GOM Niguarda, Milan, Italy; 5 Medical Library, Vrije Universiteit Amsterdam, Amsterdam, the Netherlands

**Keywords:** Node-positive, Pancreatic adenocarcinoma, Pancreatoduodenectomy, R1, Margin Status

## Abstract

**Background:**

Microscopic residual disease (R1)and node-positive disease in resected pancreatic ductal adenocarcinoma (PDAC) are associated with worse survival. Some have suggested that obtaining an R0 resection is no longer relevant when patients have node-positive disease. A meta-analysis confirming this hypothesis is lacking.

**Patients and Methods:**

A systematic review including studies with at least 50 patients with resected node-positive PDAC was conducted in PubMed, Embase, and Web of Science (inception to March 2025). Patients with node-negative disease were excluded. Primary outcome was overall survival (OS), comparing R0N+ and R1N+ resections. Hazard ratios (HR) with 95% confidence intervals (CI) represented outcome measures. Subgroup analysis included: application of the Royal College of Pathology protocol (RCP: standardized margin inking, axial slicing, and “1-mm rule”) and receipt of neoadjuvant/adjuvant therapy.

**Results:**

Overall, 19,206 patients with resected node-positive PDAC from 16 retrospective studies were included. Most patients underwent upfront resection (96.3%), primarily pancreatoduodenectomy (84.2%). The overall R0N+ rate was 61.1%, differing significantly by application of RCP (yes [R1 = tumor within 1 mm of margin]: nine studies, 52.1%; unclear: four studies, 80.5%; no [R1 = tumor at margin ink only]: three studies, 61.5%; *p* = 0.0019). Overall, R1 negatively impacted OS (HR: 1.38; 95% CI: 1.21–1.59). RCP-only subgroup analysis confirmed worse OS in R1N+ resections (HR 1.25; 95% CI 1.10–1.42). Subgroup analysis by neoadjuvant/adjuvant therapy was not feasible.

**Conclusions:**

Among patients with node-positive PDAC treated with upfront surgery, R1 resection remains associated with worse survival. In these patients, R0 resection should not be dismissed as a viable treatment goal until stronger RCP-compliant evidence emerges.

**Supplementary Information:**

The online version contains supplementary material available at https://doi.org/10.1245/s10434-026-19850-4.

Patients with pancreatic ductal adenocarcinoma who have microscopic residual disease (R1: cancer cells within 1 mm of a margin and specimen surface) following resection experience decreased overall survival (OS).^1,2^ The median OS for R1-resected pancreatic adenocarcinoma typically ranges from 14 to 21 months, whereas OS for R0 resections ranges from 23 to 28 months.^3,4^

Some authors have suggested that achieving negative resection margins (R0 > 1 mm) does not improve survival in patients with node-positive resected pancreatic adenocarcinoma at definitive pathology.^5^ Although identifying node positivity in the preoperative setting is challenging,^6^ it would change the view on the relevance of obtaining nodal status prior to resection.^7^ However, margin status may depend more on standardization, quality of specimen assessment by pathologists,^3,4,8^ and tumor biology rather than as an indicator of surgical success. Indeed, node-positive disease may represent a more advanced disease stage where systemic tumor control outweighs local control efforts. These findings challenge the traditional emphasis on surgical radicality and suggest the need for a nuanced approach to resection goals in patients with pancreatic cancer, incorporating neoadjuvant strategies that reliably lower both R1 rates—similarly with chemoradiation and chemotherapy—and nodal involvement, with chemoradiation achieving more ypN0 than chemotherapy alone.^9-11^

A meta-analysis synthesizing current data is lacking but could provide valuable insights into the prognostic and therapeutic implications of margin status in nodal-positive resected pancreatic adenocarcinoma.

## Patients and Methods

### Search Strategy

The systematic review protocol was registered on the International Prospective Register of Systematic Reviews (PROSPERO) (ID: CRD42024573155). The Preferred Reporting Items for Systematic Reviews and Meta-Analyses (PRISMA) and Assessing the Methodological Quality of Systematic Reviews (AMSTAR II) guidelines^12,13^ were followed. The PubMed, EMBASE, and Web of Science databases were screened without time restrictions from inception to 15 March 2025. The research also included all available Medical Subject Headings (MeSH) terms. The full search queries are available in the Supplementary Materials.

Articles for which free full text was unavailable were searched through the University of Milan digital library, the “Alberto Malliani” library, or direct contact with the authors. A hand search of references of included studies and previous reviews on the topic was also performed to include additional relevant studies according to our selection criteria. Two investigators (A.M.B. and S.G.) carried out the literature search independently.

### Eligibility

A specific Population (P), Intervention (I), Comparator (C), Outcome (O), and Study Design (S) (PICOS) framework was specified to define study eligibility, as recommended.

In detail, the framework was defined as follows: Population (P): patients suffering from pancreatic ductal adenocarcinoma with nodal involvement at final pathology (pN+) undergoing surgical resection; Intervention (I): patients with positive margins at final pathology at any site (R1); Comparison (C): patients with negative margins at final pathology (R0); Outcomes (O): overall survival, disease-free survival, and 90 day mortality; Study Design (S): all study designs.

Studies with the following criteria were not eligible: insufficient reporting of the PICOS criteria; studies enrolling less than 50 patients (a cutoff arbitrarily chosen to include major surgical series from referral centers); studies enrolling patients with tumors other than adenocarcinomas; studies enrolling patients with extra-regional lymph node metastases; studies enrolling pN0 patients without reporting clearly separated outcomes; overlapping series; non-English language papers; case reports, editorials, abstracts, unpublished studies, book chapters, and commentaries; and previously published reviews.

### Systematic Review Process and Data Extraction

Overall, 10,817 studies were preliminarily identified by the literature search. After excluding duplicates, two independent reviewers (A.B. and S.G.) screened titles and abstracts of 7766 studies using an a priori-developed screening form. Investigators were blinded to each other’s decisions. Two authors (A.M.B. and R.M.M.) extracted data independently. Information about study design and methodology, participant demographics and baseline characteristics, and survival outcomes were gathered in a computerized spreadsheet (Microsoft Excel 2016; Microsoft Corporation, Redmond, WA, USA). In case of disagreement, two further investigators (E.G. and M.G.B.) helped resolve discrepancies through discussion.

### Assessment of Risk of Bias and Certainty of Evidence

The risk of bias was assessed for individual studies according to the Risk of Bias in Non-Randomized Studies-of Interventions (ROBINS-I) tool provided by the Cochrane Collaboration.^14^ Data were collected according to the methodology proposed by Higgins^15^ in a computerized spreadsheet. Bar and traffic light plots were created to display the results of the risk of bias assessment graphically. Grading of Recommendations Assessment, Development and Evaluation (GRADE) recommendations were applied accordingly. Further details about are reported in the Supplementary Materials.

### Primary Outcome

The primary outcome was to assess the difference between R1N+ versus R0N resections on overall survival (OS). Subgroup analysis examined the use of a standardized pathological margin assessment protocol—either the Royal College of Pathologists (RCP) protocol or local equivalents (RCP-like)—incorporating standardized margin inking, axial slicing, and the “1-mm rule” (tumor within 1 mm of an inked margin = R1), defining three categories:

- Yes: Study reported systematic use of RCP or RCP-like protocols for all included cases.
- No: Study explicitly stated that RCP/RCP-like protocols were not used and classified R1 only when tumor was present at the inked margin.
- Unclear: Study did not specify the method of margin assessment.

Further subgroup analysis included routine versus selective intraoperative frozen section (IOFS) use and receipt of neoadjuvant/adjuvant therapy.

Secondary endpoints included disease-free survival (DFS), pattern of recurrence (local versus distant), and safety outcomes (perioperative morbidity and mortality) between R1N+ and R0N+ resections.

### Statistical Analysis

Hazard ratios (HRs) and 95% confidence intervals (CIs) represented the primary outcome measure. HR with 95% CI, and odds ratio (OR) with 95% CI represented the secondary outcome measures. Random-effects models based on the generic inverse variance method with a restricted maximum-likelihood estimator (REML) and Hartung–Knapp variance correction were developed. Between-study heterogeneity was explored using *I*^2^ statistic, but since it heavily depends on the precision of the included studies, *τ*^2^ and 95% prediction intervals were estimated as well. The presence of outliers was explored, and influential cases were detected through the leave-one-out method or graphical display of study heterogeneity (GOSH) analysis. Subgroup analyses were prespecified and performed whenever feasible to explore potential sources of heterogeneity among categorical variables. In the presence of further unexplained heterogeneity, meta-regression models were developed with continuous factors whenever feasible, using *R*^2^ to estimate the amount of variance each variable accounted for (the higher the *R*^2^ percentage value, the higher the variance explained by a particular continuous variable). HRs were retrieved from each manuscript, and their standard errors (SE) were computed from the reported 95% confidence intervals (95% CIs). If not overtly reported, they were estimated using the method proposed by Liu et al.^16^ HR and SE logarithmic transformation were derived to estimate treatment effects. Funnel plots were developed to explore publication bias, and Egger’s test of the intercept was used to quantify funnel plots’ asymmetry. Statistical analysis was conducted with R statistical software (The Comprehensive R Archive Network [CRAN], version 4.0.0 x64),^17^ using “meta,” “metafor,” “robvis,” and “dmetar” packages.Fare clic o toccare qui per immettere il testo.^18-21^

## Results

### Baseline Characteristics

**Fig. 1:**
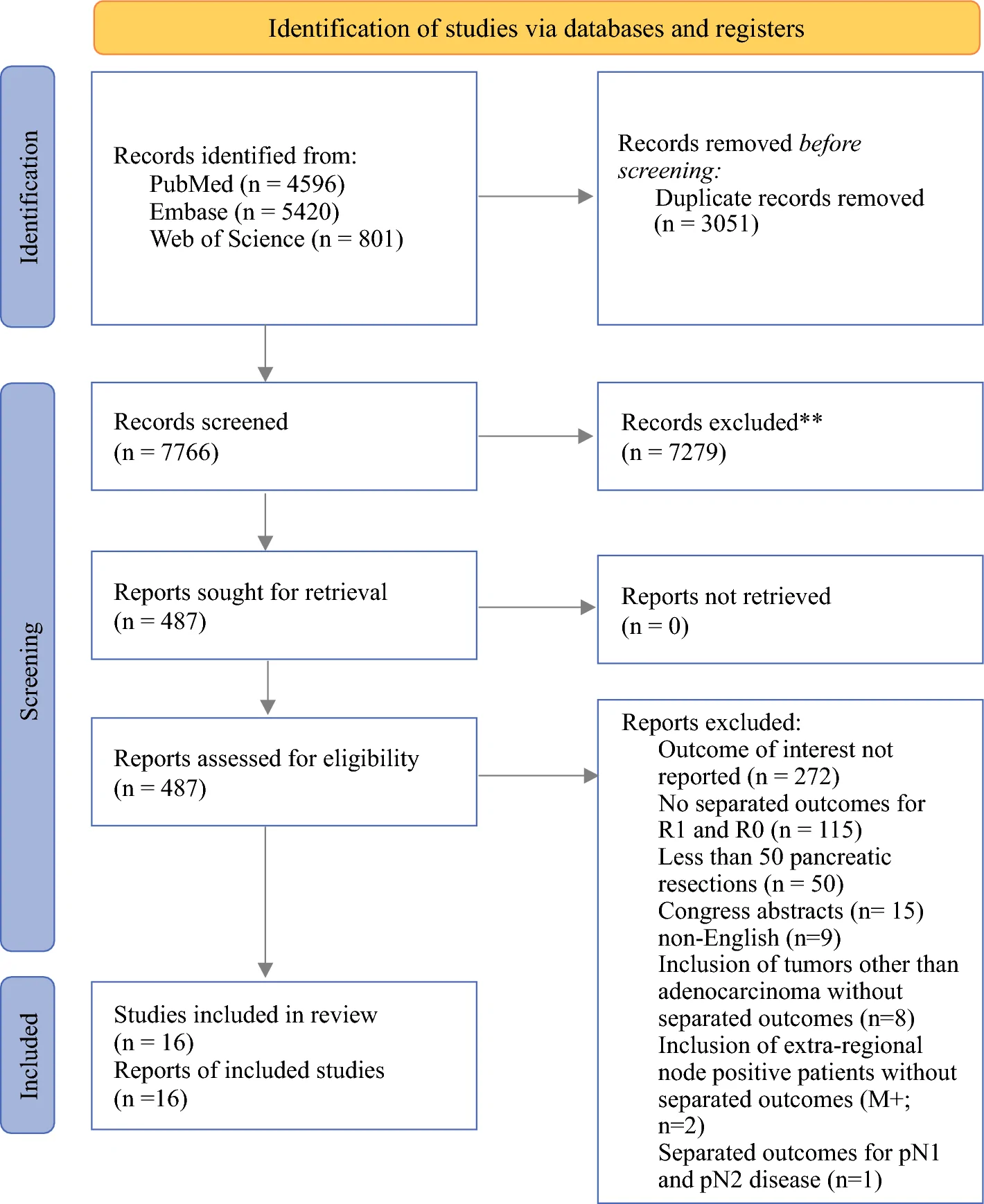
PRISMA flowchart diagram

**Fig. 2:**
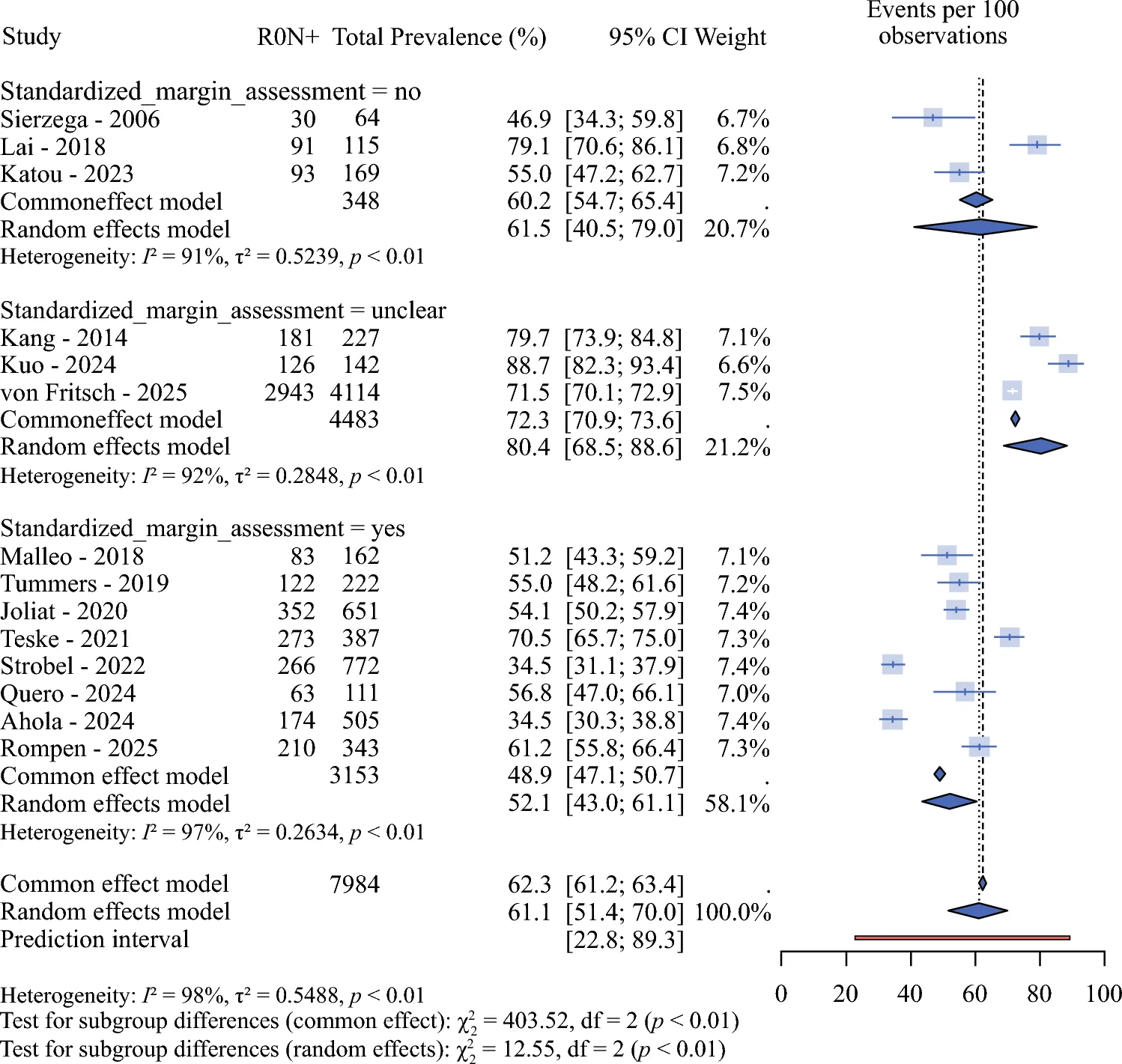
R0 rate in node-positive patients according to application of standardized margin status assessment (i.e., RCP or RCP-like). Yes = RCP or RCP-like protocol. Unclear = Method for margin assessment was not specified; it could be inferred that standardized margin assessment was not systematically applied to every case included in the study. No = Related to failure to apply the RCP protocol or principles—i.e., standardized margin inking, axial slicing, and 1-mm rule; R1 = tumor at margin ink only

Overall, 19,206 patients with resected node-positive pancreatic adenocarcinoma were included from 16 retrieved retrospective studies^5,6,22-35^ (Fig. 1). Characteristics of included studies are reported in Table 1. Most patients underwent upfront resection (96.3%; 12 studies, *n* = 3378/3505), primarily pancreatoduodenectomy (84.2%; 8 studies, *n* = 1980/2349). The overall R0N+ rate was 61.1% and differed significantly by application of RCP or RCP-like protocols (yes [RCP, i.e., R1 = tumor within 1 mm of margin]: nine studies, 52.1%; unclear: four studies, 80.5%; no [R1 = tumor at margin ink only]: three studies, 61.5%; *p* = 0.0019), with marked heterogeneity that remained high within all subgroups (Fig. 2). Perioperative morbidity and mortality were reported in only two studies, but these did not provide separate data for R1 and R0 resections. The rate of adjuvant therapy delivery was approximately 60%, with only two studies reporting the treatment regimen, which was predominantly gemcitabine-based. Preoperative carbohydrate antigen (CA) 19-9 values, tumor size, patient performance status, and resectability status were not available. Further general characteristics of included node-positive patients are reported in Table 2.

**Table 1:** Characteristics of included studies

Author (year)	NOS	RoB	Country	Routine IOFS	Standardized margin assessment	N+ sample size (*n*)	HR (95% CI)^b^	Retrieval of HR	OS
Sierzega (2006)	6	Serious	Poland	Unclear^a^	No	64	9.40 (2.23–39.53)	Multivariable	R1 < R0
Kang (2014)	7	Low	Korea	Unclear^a^	Unclear^a^	227	1.66 (0.92–3.01)	Multivariable	R1 = R0
Delpero (2017)	7	Low	France	Yes	Yes (RCP)	93	1.3 (0.7–2.3)	Multivariable	R1 = R0
Tran Cao (2017)	6	Moderate	USA	Unclear^a^	Unclear^a^	11129	1.47 (1.40–1.54)	Multivariable	R1 < R0
Lai (2018)	8	Moderate	Taiwan	Unclear^a^	No	115	0.67 (0.34–1.4)	Multivariable	R1 = R0
Malleo (2018)	7	Moderate	Italy and USA	Unclear^a^	Yes (RCP-like)	162	1.13 (0.77–1.64)	Univariate	R1 = R0
Tummers (2019)	8	Moderate	Netherlands	Unclear^a^	Yes (RCP-like)	222	1.26 (0.92–1,73)	Kaplan–Meier	R1 = R0
Joliat (2020)	8	Moderate	International	Yes	Yes (RCP)	651	1.10 (0.80–1.40)	Multivariable	R1 = R0
Teske (2021)	9	Low	Germany	Yes	Yes (RCP-like)	387	1.27 (0.90–1.79)	Kaplan–Meier	R1 = R0
Strobel (2022)	9	Moderate	Germany	Unclear^a^	Yes (RCP-like)	772	1.19 (1.02–1.39)	Kaplan–Meier	R1 < R0
Katou (2023)	7	Moderate	Germany and Switzerland	yes	No	169	1.56 (1.15–2.13)	Kaplan–Meier	R1 < R0
Kuo (2024)	7	Low	USA	Unclear^a^	Unclear^a^	142	2.64 (1.51–4.63)	Multivariable	R1 < R0
Quero (2024)	7	Moderate	Italy	Yes	Yes (RCP)	111	1.33 (0.81–2.17)	Kaplan–Meier	R1 = R0
Ahola (2024)	9	Low	International	Yes	Yes (RCP)	505	1.04 (0.72–1.51)	Univariate	R1 = R0
Rompen (2025)	9	Moderate	USA	51% of patients	Yes (RCP-like)	343	1.84 (1.37–2.48)	Kaplan–Meier	R1 < R0
von Fritsch (2025)	7	Moderate	Germany	Unclear^a^	Unclear^a^	4114	1.56 (1.37–1.77)	Kaplan–Meier	R1 < R0

^a^The study did not detail the method for margin assessment or IOFS use; it could be inferred that neither standardized margin assessment nor IOFS was systematically applied to every case included in the study. ^b^Hazard ratio (HR; 95% CI): expressed as R1 over R0 resection, with R0 as the reference; HR > 1 indicates an increased risk of death, while HR < 1 indicates a decreased risk. The HR is not significant if the 95% confidence interval crosses 1. *NOS* Newcastle–Ottawa Scale,^10^ *RoB* risk of bias, *IOFS* intraoperative frozen section (with subsequent re-resection in case of positivity), *OS* overall survival, *RCP* Royal College of Pathology protocol, *RCP-like* national protocol following principles firstly described in the Royal College of Pathology protocol

**Table 2:** Baseline characteristics of patients with node-positive resected pancreatic adenocarcinoma

Characteristic	Percentage (*n*/total patients)
Neoadjuvant therapy	3.4% (12 studies, *n* = 127/3505)
*Surgery*	
Pancreatoduodenectomy	84.2% (8 studies, *n* = 1980/2349)
Left pancreatectomy	10.8% (8 studies, *n* = 255/2349)
Total pancreatectomy	4.6% (8 studies, *n* = 108/2349).
Venous resection	17.8% (5 studies; *n* = 334/1874)
Arterial resection	2% (5 studies; *n* = 39/1874)
*Pathology*	
pT3–T4	64.2% (4 studies; *n* = 992/1545)
Poorly differentiated (G3)	40.8% (6 studies; *n* = 595/1457)
pN2	45.2% (4 studies; *n* = 624/1378)
Adjuvant therapy	59.3% (6 studies; *n* = 1196/2016)

### Primary Endpoint

**Fig. 3:**
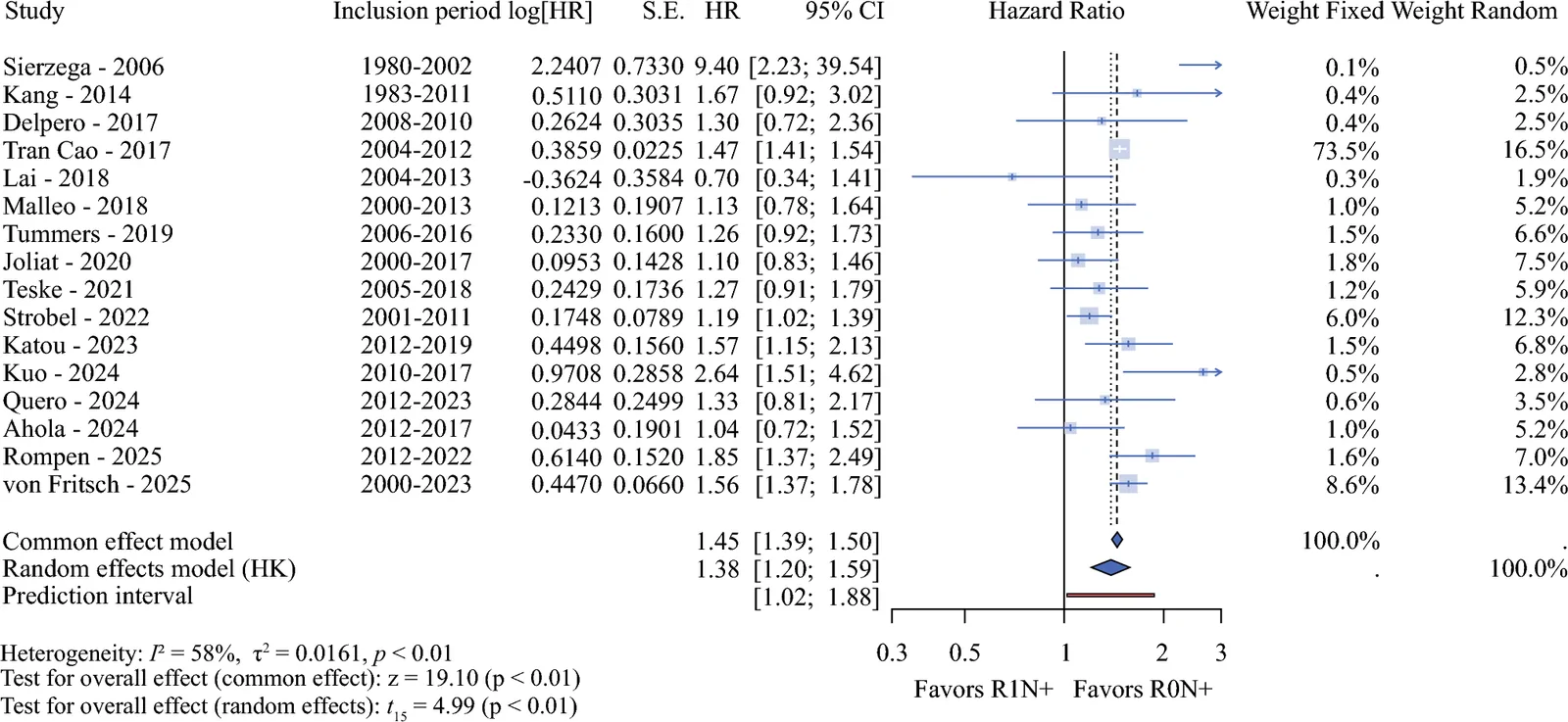
Forest plot for overall survival before exclusion of outliers

All studies reported data regarding OS. Only one study clearly included 90-day mortality in survival analysis. Patients with R1 at final pathology experienced a worse prognosis (HR: 1.38; 95% CI: 1.21–1.59; *p* = 0.0002; Fig. 2). Moderate, significant heterogeneity was detected (*I*^2^ = 58.3%; *τ*^2^ = 0.016; prediction interval [PI]: 1.02–1.88; *Q*-test *p* = 0.0018). The study by Sierzega et al.^29^ was identified as an outlier by both visual inspection of the plot (the lower bound of its 95% CI exceeded the upper bound of the pooled 95% CI) and R output. After excluding it, worse OS for patients with R1 was confirmed (HR: 1.37; 95% CI: 1.21–1.55; *p* < 0.0001; Fig. 3), and heterogeneity remained moderate (*I*^2^ = 52.5%; *τ*^2^ = 0.052; PI: 1.02–1.83; *Q*-test *p* = 0.009). The forest plot after exclusion of outliers is shown in the Supplementary Materials (Supplementary Fig. S1).

### Subgroup Analysis

Subgroup analysis for application of a standardized protocol of pathological examination (i.e., RCP or following RCP principles)^3^ confirmed a worse prognosis for patients with a R1 resection (nine studies; HR: 1.25; 95% CI: 1.10–1.42), as compared with an R0 resection. Heterogeneity was low (*I*^2^ = 12%; *τ*^2^ = 0.0064; PI: 0.99–1.58; *Q*-test *p* = 0.34), with most of the observed heterogeneity attributable to the remaining two subgroups (unclear or no application of a standardized margin assessment; Table 3 and Supplementary Figs. S2 and S3). Subgroup analysis for routine versus non-routine IOFS use during surgery highlighted that R1 resection was associated with worse survival regardless of routinely performed IOFS (Tables 2 and 3 and Supplementary Figs. S4 and S5). Notably, no study omitted IOFS use.

**Table 3:** Subgroup analysis for impact of R1 resection on overall survival in patients with standardized margin assessment and IOFS use

Variable		HR (95% CI)^c^	*I*^2^ (%)	*τ*^2^	*Q*-test for heterogeneity	Test for subgroup differences (*p*-Value)
Standardized margin assessment (i.e., RCP or RCP-like)	No	1.11 (0.01–181.84)	76.8	0.25	0.04	0.025
	Unclear^b^	1.5 (1.32–1.72)	39.8	0.001	0.17	
	Yes	1.25 (1.1–1.42)	11.6	0.006	0.34	
Routine IOFS^a^	Unclear^b^	1.39 (1.15–1.68)	63.6	0.02	< 0.01	0.49
	Yes	1.29 (1.07–1.56)	0	0.002	0.59	

^a^No study omitted IOFS. ^b^Method for margin assessment or routine IOFS use was not specified; it could be inferred that neither standardized margin assessment nor IOFS was systematically applied to every case included in the study. ^c^Hazard ratio (HR; 95% CI): expressed as R1 over R0 resection, with R0 as the reference; HR > 1 indicates an increased risk of death, while HR < 1 indicates a decreased risk. The HR is not significant if the 95% confidence interval crosses 1

Subgroup analysis for receipt of neoadjuvant^6,22,25,26,31,34^ or adjuvant therapy^23,25,26,31,32^ was not feasible owing to missing data.

### Secondary Endpoints

Five studies reported data regarding DFS. No difference in DFS was detected between R1N+ and R0N+ patients (HR: 1.21; 95% CI: 0.99–1.49; *p* = 0.059). Heterogeneity was low (*I*^2^ = 8%; *τ*^2^ = 0; PI: 0.95–1.54; *Q*-test *p* = 0.36). The forest plot for DFS is depicted in Supplementary Fig. S6 in the Supplementary Materials. No study reported 90-day mortality and pattern of recurrence for R1N+ versus R0N, making meta-analysis for these secondary outcomes unfeasible.

### Meta-Regression

Year of publication, sample size, proportion of advanced T (T3–4), N (≥ N2) stage, and poorly differentiated grading (G3) were entered in meta-regression model one by one. None of these variables were significantly associated with the effect size (*R*^2^ = 0 for each continuous variable). Detailed results are reported in Supplementary Table S1 in the Supplementary Materials.

### Risk of Bias, Publication Bias

**Fig. 4:**
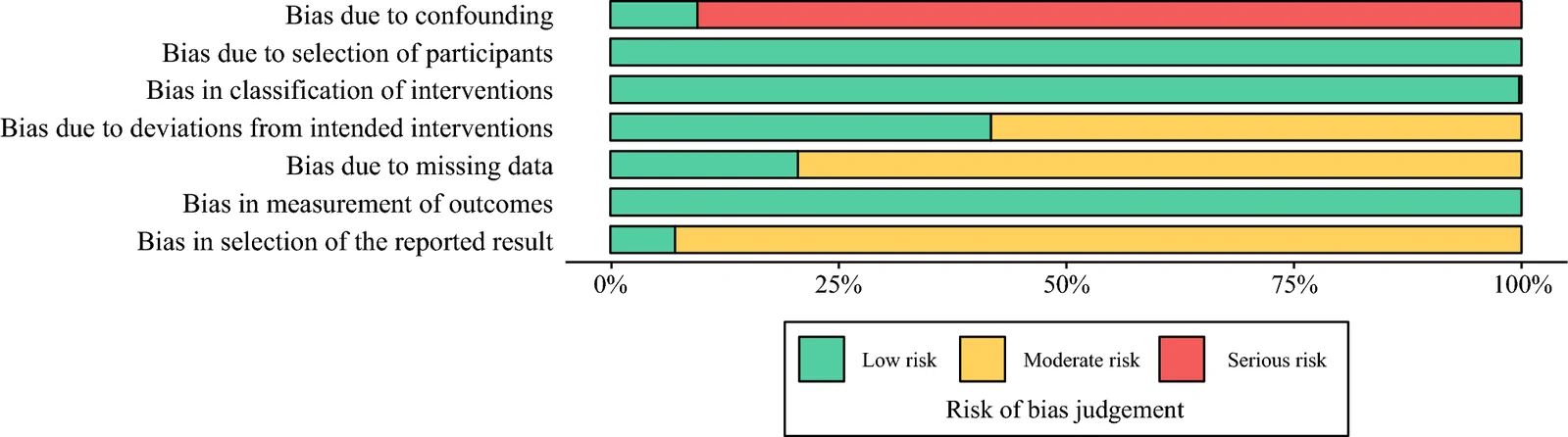
Bar plot of risk of bias

Risk of bias for each individual study is reported in Table 1. The results of the RoB assessment are shown in Fig. 4 and Supplementary Fig. S7 in the Supplementary Materials. Egger’s test of the intercept (OS: *p* = 0.62; DFS: *p* = 0.5) and funnel plot inspection failed to identify publication bias for any outcome. The contour-enhanced funnel plot is reported in the Supplementary Materials (Supplementary Fig. S8).

### Certainty of Evidence

The overall certainty of evidence was low. Inconsistency was considered serious owing to the lack of similarity of the point estimates. Imprecision was judged as serious owing to the wide width of the 95% CI in half of the studies. These factors contributed to the decrease in certainty of evidence. A detailed assessment is reported in Supplementary Fig. S9 in the Supplementary Materials.

## Discussion

This systematic review and meta-analysis involving 19,206 patients (16 studies) confirmed that also in node-positive upfront resected pancreatic adenocarcinoma, R1 resection increased the risk of death by 38% (HR: 1.38; Fig. 2), as compared with R0 resection. Subgroup analysis in studies using the RCP (or RCP-like) standardized method for margin assessment^3^ confirmed this statistically and clinically significant association (HR: 1.25; Supplementary Fig. S2), allowing for the conclusion that microscopic margin clearance (R0 resection > 1 mm) is associated with improved survival also in patients with node-positive upfront resected pancreatic adenocarcinoma.^36^

Our findings also underscore the common saying that “biology is king” in resections of node-positive pancreatic adenocarcinoma. First, the R0 resection rate in our cohort was lower (61.1%) compared with the most recent meta-analysis of resected patients with pancreatic ductal adenocarcinoma (PDAC) (irrespective of nodal status), which reported an R0 rate of 67.5%.^36^ Additionally, R1 was not clearly associated with worse DFS (HR 1.21; 95% CI 0.99–1.49; Supplementary Fig. S6 in the Supplementary Materials), possibly indicating that a lower proportion of both R0 resections and disease control is achieved when nodal involvement is present. Micrometastatic spread may already be present even after an R0 resection,^37^ especially considering node positivity.^38^

Second, heterogeneity of the primary outcome seemed tied to both non-standardized margin assessment (*I*^*2*^ = 76.8%, *τ*^2^ = 0.253; Table 3) and unclear/inconsistent IOFS use (*I*^2^ = 63.6%; *τ*^2^ = 0.02; Table 3) and not to tumor characteristics, center surgical volume, or year of resection (Supplementary Table S1). Additionally, the R0 resection rate was significantly higher with non-standardized margin assessment compared with specimens assessed with RCP protocol (importantly, Fig. 2 shows wide variation in R1 rates, with broad confidence intervals and substantial heterogeneity despite all cohorts including patients with the same condition—i.e., node-positive PDAC undergoing upfront resection). These findings may suggest that even the most successful and technically sound surgery, including careful intraoperative frozen section analysis in a high-volume setting, can lead to misclassified R0 resections, which would be classified as R1 resections if all specimen surfaces were included and analyzed per the RCP protocol.^39,40^ This interpretation may be supported by the variability in the impact of microscopic margin clearance on survival reported in the literature^41–44^ as well as in studies included in this analysis (Table 1 and Fig. 2), which may stem from interobserver and interinstitution variability in pathological assessment of a biologically aggressive disease,^45–47^ rather than small variations in surgical technique.

While R0 resection remains a key element of any multimodal treatment plan, as confirmed by our results, growing evidence shows that long-term survival in resected pancreatic cancer depends not only on margin clearance and anatomical resectability but also on patient status and tumor biology.^48–50^ These factors—unaddressed here owing to missing data—should be equally central in guiding both the indication for, and timing of, resection. A personalized decision-making process aimed at achieving the best possible outcome for each patient should include discussion of: (a) the use of neoadjuvant therapy for resectable tumors (chemotherapy,^9^ with consideration of adding chemoradiation^10,11^ when the aim is to increase the rate of ypN0^51^ and/or switching chemotherapy regimen in case of inadequate response^9-11,50,52^; (b) the recognition that R1 resection does not necessarily preclude long-term survival,^53,54^ but must still be regarded as a negative prognostic factor,^55^ possibly even after neoadjuvant therapy^51^; (c) the possibility that postponing or even avoiding resection altogether may represent the best option for a subset of patients (i.e., unfavorable performance, CA 19-9, and radiological profile at presentation^49,52^ and/or during neoadjuvant treatment ^50^).

Notably, the results of the current study only apply to patients with locoregional lymph nodes undergoing upfront resection, of whom 60% subsequently received adjuvant therapy. Indeed, a recent multicenter study involving 670 resected pancreatic adenocarcinomas with advanced lymph node metastasis (pN2) from German hospitals found no survival benefit of an R0 versus R1 resection.^31^ Similarly, in a recent analysis of 472 resected pancreatic adenocarcinomas at NYU, margin status was no longer a significant prognostic factor in multivariable analysis for patients with radiologically defined borderline resectable and locally advanced pancreatic cancers who underwent surgery with curative intent.^34^

Our results must be interpreted considering several limitations. First, all included studies were retrospective and most of them excluded 90-day mortality from survival analysis, introducing significant selection bias and contributing to an overall low quality of evidence (Supplementary Fig. S9). Second, only few studies reported separated outcomes for type of R1 (two studies; direct and < 1 mm) and site of R1 (three studies, noncomparable), despite evidence that they both have survival implications.^55^ Fourth, the included studies underrecognized disease burden, underused neoadjuvant therapy (precluding subgroup analysis), underreported adjuvant treatment regimens (when specified only in two studies, most were gemcitabine-based), and lack a clear definition of R2 resection. While definitions of “biological R2” are emerging,^56^ the reality may stem from inadequacy of surgical reports—some R1 resections with tumor at the margin ink may, in fact, be R2 resections—and insufficient staging of surgical candidates.^57-59^ Fifth, the quality of the hazard ratios (univariate or derived from Kaplan–Meier curves) warrants caution in interpreting our results (Table 1). Sixth, the HR of DFS approached statistical significance in favoring R0 over R1 resections (Fig. 2). Finally, the absence of intention-to-treat study designs (only one study incorporated perioperative mortality into the survival analysis, which is particularly relevant if one assumes that pursuing an R0 resection may be associated with higher 90-day mortality) and missing data of patient and tumor-related characteristics (including performance status, CA 19-9, tumor size, resectability status, type and site of R1, adjuvant therapy regimen, and recurrence treatment) may have led to an overestimation of the survival benefit of R0 resection in our study population. Importantly, despite being statistically significant, an HR of 1.25 true effect size may not be considered clinically significant outside of the population of this study.^60^ The strength of the present study lies in its rigorous inclusion criteria, which helped mitigate an otherwise moderate-to-high risk of bias (Table 1 and Fig. 2) and contributed to the statistical robustness of the primary outcome (Fig. 2). The use of OS as the primary outcome, rather than other surrogate markers, lends greater clinical validity to these findings.

In conclusion, R1 resection is associated with worse survival also in node-positive upfront resected pancreatic adenocarcinoma. Despite the overall low quality of evidence and the limited use of neoadjuvant and possibly even contemporary adjuvant regimens,^60,61^ which restrict the applicability of these findings to the current treatment landscape, efforts to achieve an R0 resection should not be dismissed as a clinically significant positive prognostic factor for these patients until higher-quality evidence becomes available. Importantly, our study highlights that margin assessment following RCP or RCP-like protocol (standardized margin inking, axial slicing, and 1-mm rule) remains somewhat underused (only 9 out of 16 studies), challenging (Fig. 2), and crucial to avoid misclassification of margin status: some R1s identified with RCP or RCP-like protocol may in fact be considered R0s if margin were to be addressed without respecting RCP sites and principles, skewing true R0s survival outcomes. Assuming optimal timing of resection, surgical technique, and pathological assessment, further studies with intention-to-treat design, adequate follow-up, and thorough reporting of systemic therapy regimen^60,61^ and recurrence treatment^62^ are needed to clarify: (a) the impact of margin status on survival of resected pancreatic ductal adenocarcinoma after neoadjuvant therapy and possibly after contemporary adjuvant regimens and (b) whether an R1 resection is justified over starting or continuing systemic therapy.

## Supplementary Information

Below is the link to the electronic supplementary material.

Supplementary file1 (DOCX 2238 KB)
