# Supplementary material for: Impact of R0/R1 Margin Status on Overall Survival in Node-Positive Resected Pancreatic Ductal Adenocarcinoma: Meta-Analysis
Source: Ann Surg Oncol. 2026 Jul 8;33(10):9361–71. doi: 10.1245/s10434-026-19850-4 (PMC13586057; doi:10.1245/s10434-026-19850-4)
Supplement: Supplementary file 1 [file 10434_2026_19850_MOESM1_ESM.docx]

**Impact of R0/R1 margin status on overall survival in node-positive resected pancreatic ductal adenocarcinoma: meta-analysis**

SUPPLEMENTARY MATERIALS

| Search query | 2 |
| --- | --- |
| Assessment of risk of bias and certainty of evidence | 6 |
| Forest plot OS (Figure S1) | 7 |
| Forest plot OS before exclusion of outliers with subgroups for margin assessment (Figure S2, S3) | 8 |
| Forest plot OS before and after with subgroups for IOFS use (Figure S4, Figure S5) | 10 |
| Forest plot DFS (Figure S6) | 12 |
| Risk of Bias - traffic light plot (Figure S7) | 13 |
| Funnel plots of publication bias (Figure S8) | 14 |
| GRADE (Figure S9) | 15 |
| Metaregression (Figure S11) | 16 |

**Search query**

The search strategy included controlled vocabulary and free-text terms, including synonyms and closely related words, of ‘Pancreatic Neoplasms,’ ‘Lymphatic Metastasis,’ ‘Pancreatectomy’ or ‘Margins of Excision,’ and ‘Prognosis,’ ‘Survival Rate,’ or ‘Overall Survival.’ These terms were combined with synonyms for randomized controlled trials (RCTs) and cohort studies. No restrictions were applied regarding language or publication date.

Duplicate articles were excluded by the medical information specialist (AV-I) using Endnote X20.0.1 (Clarivate™), following the Amsterdam Efficient Deduplication (AED) method [[Reference](https://search.datacite.org/works/10.5281/zenodo.4544315)] and the Bramer method [2]. No grey literature sources were consulted in this review.

*References*

*[1] René Otten, Ralph de Vries, & Linda Schoonmade. (2019). Amsterdam Efficient Deduplication (AED) method (Versione 1). Zenodo.* [*https://doi.org/10.5281/zenodo.4544315*](https://doi.org/10.5281/zenodo.4544315)	*[2] Bramer WM, Giustini D, de Jonge GB, Holland L, Bekhuis T. De-duplication of database search results for systematic reviews in EndNote. J Med Libr Assoc. 2016 Jul;104(3):240-3. doi: 10.3163/1536-5050.104.3.014. Erratum in: J Med Libr Assoc. 2017 Jan;105(1):111. doi: 10.5195/jmla.2017.128. PMID: 27366130; PMCID: PMC4915647.*

1) PubMed

| **Search** | **Query** |
| --- | --- |
| #8 | Search: **#5 AND (#6 OR #7)**  ("Pancreatic Neoplasms"[MeSH Terms] OR "carcinoma, pancreatic ductal"[MeSH Terms] OR "Adenocarcinoma"[MeSH Terms] OR "PDAC"[Title/Abstract] OR ("pancre*"[Title/Abstract] AND ("adenocarcino*"[Title/Abstract] OR "carcino*"[Title/Abstract]))) AND ("Lymph Nodes"[MeSH Terms] OR "Lymphatic Metastasis"[MeSH Terms] OR "Neoplasm Staging"[MeSH Terms] OR "n sta*"[Title/Abstract] OR "N0"[Title/Abstract] OR "N1"[Title/Abstract] OR (("lymph*"[Title/Abstract] OR "lymphonodular"[Title/Abstract]) AND ("positive"[Title/Abstract] OR "metast*"[Title/Abstract]))) AND ("Pancreatectomy"[MeSH Terms] OR "Margins of Excision"[MeSH Terms] OR "microscopic residual tumo*"[Title/Abstract] OR "R1"[Title/Abstract] OR "R0"[Title/Abstract] OR ("margin*"[Title/Abstract] AND ("excis*"[Title/Abstract] OR "resec*"[Title/Abstract]))) AND ("Prognosis"[MeSH Terms] OR "Survival Rate"[MeSH Terms] OR "Mortality"[MeSH Terms] OR "Disease-Free Survival"[MeSH Terms] OR "overall surviv*"[Title/Abstract] OR "5YS"[Title/Abstract] OR "3YS"[Title/Abstract] OR "5 year surv*"[Title/Abstract] OR "3 year surv*"[Title/Abstract] OR "OS"[Title/Abstract] OR "five year surv*"[Title/Abstract] OR "three year surv*"[Title/Abstract]) AND ("Epidemiologic Studies"[MeSH Terms] OR "cohort"[Title/Abstract] OR ("case"[Title/Abstract] AND ("control"[Title/Abstract] OR "controll*"[Title/Abstract] OR "comparison"[Title/Abstract] OR "referent"[Title/Abstract])) OR "risk"[Title/Abstract] OR "causation"[Title/Abstract] OR "causal"[Title/Abstract] OR "odds ratio"[Title/Abstract] OR "etiol*"[Title/Abstract] OR "aetiol*"[Title/Abstract] OR "natural history"[Title/Abstract] OR "predict*"[Title/Abstract] OR "prognos*"[Title/Abstract] OR "outcome"[Title/Abstract] OR "course"[Title/Abstract] OR "retrospect*"[Title/Abstract] OR (("random*"[Title/Abstract] AND ("controlled"[Title/Abstract] OR "control"[Title/Abstract] OR "placebo"[Title/Abstract] OR "versus"[Title/Abstract] OR "vs"[Title/Abstract] OR "group"[Title/Abstract] OR "groups"[Title/Abstract] OR "comparison"[Title/Abstract] OR "compared"[Title/Abstract] OR "arm"[Title/Abstract] OR "arms"[Title/Abstract] OR "crossover"[Title/Abstract] OR "cross-over"[Title/Abstract]) AND ("trial"[Title/Abstract] OR "study"[Title/Abstract])) OR (("single"[Title/Abstract] OR "double"[Title/Abstract] OR "triple"[Title/Abstract]) AND ("masked"[Title/Abstract] OR "blind*"[Title/Abstract])))) |
| #7 | Search: **((random*[tiab] AND (controlled[tiab] OR control[tiab] OR placebo[tiab] OR versus[tiab] OR vs[tiab] OR group[tiab] OR groups[tiab] OR comparison[tiab] OR compared[tiab] OR arm[tiab] OR arms[tiab] OR crossover[tiab] OR cross-over[tiab]) AND (trial[tiab] OR study[tiab])) OR ((single[tiab] OR double[tiab] OR triple[tiab]) AND (masked[tiab] OR blind*[tiab])))** |
| #6 | Search: **"Epidemiologic Studies"[Mesh] OR cohort[tiab] OR (case[tiab] AND (control[tiab] OR controll*[tiab] OR comparison[tiab] OR referent[tiab])) OR risk[tiab] OR causation[tiab] OR causal[tiab] OR "odds ratio"[tiab] OR etiol*[tiab] OR aetiol*[tiab] OR "natural history"[tiab] OR predict*[tiab] OR prognos*[tiab] OR outcome[tiab] OR course[tiab] OR retrospect*[tiab]** |
| #5 | Search: **#1 AND #2 AND #3 AND #4** |
| #4 | Search: **"Prognosis"[Mesh] OR "Survival Rate"[Mesh] OR "Mortality"[Mesh] OR "Disease-Free Survival"[Mesh] OR "overall surviv*"[tiab] OR "5YS"[tiab] OR "3YS"[tiab] OR "5-year surv*"[tiab] OR "3-year surv*"[tiab] OR "OS"[tiab] OR "Five-year surv*"[tiab] OR "three-year surv*"[tiab]** |
| #3 | Search: **"Pancreatectomy"[Mesh] OR "Margins of Excision"[Mesh] OR "Microscopic residual tumo*"[tiab] OR R1[tiab] OR R0[tiab] OR (Margin*[tiab] AND (Excis*[tiab] OR Resec*[tiab]))** |
| #2 | Search: **"Lymph Nodes"[MeSH Terms] OR "Lymphatic Metastasis"[MeSH Terms] OR "Neoplasm Staging"[MeSH Terms] OR "n sta*"[tiab] OR "N0"[tiab] OR "N1"[tiab] OR (("lymph*"[tiab] OR "lymphonodular"[tiab]) AND (positive[tiab] OR "metast*"[tiab]))** |
| #1 | Search: **"Pancreatic Neoplasms"[Mesh] OR "Carcinoma, Pancreatic Ductal"[Mesh] OR "Adenocarcinoma"[Mesh] OR PDAC[tiab] OR (Pancre*[tiab] AND (Adenocarcino*[tiab] OR Carcino*[tiab]))** |

2) Embase

| Search | Query |
| --- | --- |
| #10 | **#8 NOT #9** |
| #9 | **#8 AND ('conference abstract'/it OR 'conference paper'/it OR 'conference review'/it)** |
| #8 | **#5 AND (#6 OR #7)** |
| #7 | **(('random*':ti,ab,kw AND ('controlled':ti,ab,kw OR 'control':ti,ab,kw OR 'placebo':ti,ab,kw OR 'versus':ti,ab,kw OR 'vs':ti,ab,kw OR 'group':ti,ab,kw OR 'groups':ti,ab,kw OR 'comparison':ti,ab,kw OR 'compared':ti,ab,kw OR 'arm':ti,ab,kw OR 'arms':ti,ab,kw OR 'crossover':ti,ab,kw OR 'cross-over':ti,ab,kw) AND ('trial':ti,ab,kw OR 'study':ti,ab,kw)) OR (('single':ti,ab,kw OR 'double':ti,ab,kw OR 'triple':ti,ab,kw) AND ('masked':ti,ab,kw OR 'blind*':ti,ab,kw)))** |
| #6 | **'epidemiology'/exp OR 'cohort':ti,ab,kw OR ('case':ti,ab,kw AND ('control':ti,ab,kw OR 'controll*':ti,ab,kw OR 'comparison':ti,ab,kw OR 'referent':ti,ab,kw)) OR 'risk':ti,ab,kw OR 'causation':ti,ab,kw OR 'causal':ti,ab,kw OR 'odds ratio':ti,ab,kw OR 'etiol*':ti,ab,kw OR 'aetiol*':ti,ab,kw OR 'natural history':ti,ab,kw OR 'predict*':ti,ab,kw OR 'prognos*':ti,ab,kw OR 'outcome':ti,ab,kw OR 'course':ti,ab,kw OR 'retrospect*':ti,ab,kw** |
| #5 | **#1 AND #2 AND #3 AND #4** |
| #4 | **'prognosis'/exp OR 'survival rate'/exp OR 'mortality'/exp OR 'disease free survival'/exp OR 'overall surviv*':ti,ab,kw OR '5ys':ti,ab,kw OR '3ys':ti,ab,kw OR '5-year surv*':ti,ab,kw OR '3-year surv*':ti,ab,kw OR 'os':ti,ab,kw OR 'five-year surv*':ti,ab,kw OR 'three-year surv*':ti,ab,kw** |
| #3 | **'pancreatectomy'/exp OR 'surgical margin'/exp OR 'microscopic residual tumo*':ti,ab,kw OR 'r1':ti,ab,kw OR 'r0':ti,ab,kw OR ('margin*':ti,ab,kw AND ('excis*':ti,ab,kw OR 'resec*':ti,ab,kw))** |
| #2 | **'lymph node'/exp OR 'lymph node metastasis'/exp OR 'cancer staging'/exp OR 'n sta*':ti,ab,kw OR 'n0':ti,ab,kw OR 'n1':ti,ab,kw OR (('lymph*':ti,ab,kw OR 'lymphonodular':ti,ab,kw) AND ('positive':ti,ab,kw OR 'metast*':ti,ab,kw))** |
| #1 | **'pancreas tumor'/exp OR 'pancreatic ductal carcinoma'/exp OR 'adenocarcinoma'/exp OR 'pdac':ti,ab,kw OR ('pancre*':ti,ab,kw AND ('adenocarcino*':ti,ab,kw OR 'carcino*':ti,ab,kw))** |

3) Web of Science

| Search | Query |
| --- | --- |
| #8 | **#5 AND (#6 OR #7)** |
| #7 | **TS=(((“random*” AND (“controlled” OR “control” OR “placebo” OR “versus” OR “vs” OR “group” OR “groups” OR “comparison” OR “compared” OR “arm” OR “arms” OR “crossover” OR “cross-over”) AND (“trial” OR “study”)) OR ((“single” OR “double” OR “triple”) AND (“masked” OR “blind*”))))** |
| #6 | **TS=(“cohort” OR (“case” AND (“control” OR “controll*” OR “comparison” OR “referent”)) OR “risk” OR “causation” OR “causal” OR "odds ratio" OR “etiol*” OR “aetiol*” OR "natural history" OR “predict*” OR “prognos*” OR “outcome” OR “course” OR “retrospect*”)** |
| #5 | **#1 AND #2 AND #3 AND #4** |
| #4 | **TS=(“prognosis” OR “survival rate” OR “Mortality” OR “Disease-free survival” OR "overall surviv*" OR "5YS" OR "3YS" OR "5-year surv*" OR "3-year surv*" OR "OS" OR "Five-year surv*" OR "three-year surv*")** |
| #3 | **TS=("Microscopic residual tumo*" OR “R1” OR “R0” OR (“Margin*” AND (“Excis*” OR “Resec*”)))** |
| #2 | **TS=("n sta*" OR "N0" OR "N1" OR (("lymph*" OR "lymphonodular") AND (“positive” OR "metast*")))** |
| #1 | **TS=("PDAC" OR ("Pancre*" AND ("Adenocarcino*" OR "Carcino*")))** |

**Assessment of risk of bias and certainty of evidence**

The following domains were explored to assess Risk of Bias: 1) bias due to confounding; 2) bias in selection of participants into the study; 3) bias in classification of interventions; 4) bias due to departures from intended interventions; 5) bias due to missing data; 6) bias in measurement of outcomes; 7) bias in selection of the reported results.

GRADE recommendations were applied as follows: .

- Risk of bias was assessed using the ROBINS-I tool.
- Indirectness: ROBINS-I “Bias in selection of participants into the study”, “Bias due to deviations from intended interventions”, and “Bias in measurement of the outcome” domains were used to assess applicability and surrogate outcomes concerns.
- Inconsistency was evaluated based on the similarity of the point estimates, the extent of overlap among CIs, and heterogeneity. Sensitivity analysis was conducted to detect potential sources of heterogeneity.
- Imprecision assessment was mainly based on both the width of the CI and the number of participants in the studies.
- Publication bias was assessed using funnel plot and regression test.

**Figure S1: Forest plot OS after exclusion of outliers**

**Figure S2: Forest plot for OS after exclusion of outliers with subgroups for margin status assessment**

**Figure S3: Forest plot OS before exclusion of outliers with subgroups for margin assessment**

**Figure S4: Forest plot OS after exclusion of outliers with subgroups for routine IOFS**

**Figure S5: Forest plot OS before exclusion of outliers with subgroups for routine IOFS**

**Figure S6: Forest plot for disease free survival**

**Figure S7: Risk of Bias - Traffic light plot**

**Figure S8: Funnel plots of publication bias**

**Figure S9: Certainty of evidence according to GRADE recommendations**

*Two studies (Tran Cao et. al, Delpero et. al) did not report number of patients for each subgroup
HR (95% CI): Hazard Ratio, expressed as R1 over R0 resection, with R0 as the reference; HR > 1 indicates an increased risk of death, while HR < 1 indicates a decreased risk. The HR is not significant if the 95% confidence interval (95% CI) crosses 1.

**Table S1: Metaregression**

| **Variable** | **I^2^ (%)** | **R^2^ (%)** | **p-value** | **Test for residual heterogeneity** |
| --- | --- | --- | --- | --- |
| Year of publication | 48.71 | 0 | 0.885 | **0.001** |
| Sample size | 46.69 | 0 | 0.595 | **0.003** |
| Proportion of T stage 3-4 | 0 | 0 | 0.572 | 0.469 |
| Proportion of N stage ≥2 | 70.3 | 0 | 0.867 | **0.032** |
| Proportion of G3 | 53.47 | 0 | 0.628 | 0.064 |
